# Phylogenetic position of the pigeon mite, *Ornithonyssus sylviarum*, with amplification of its immunogenetic biomarkers in Egypt

**DOI:** 10.1038/s41598-024-72433-9

**Published:** 2024-09-25

**Authors:** Mai A. Salem, Noha Madbouly Taha, Mohamed M. El-Bahy, Reem M. Ramadan

**Affiliations:** 1 Department of Parasitology, Faculty of Veterinary Medicine, Cairo University, Giza, 1221 Egypt; 2 Department of Parasitology, Faculty of Medicine, Cairo University, Giza, Egypt

**Keywords:** *Ornithonyssus sylviarum*, Pigeon, Genotyping, Cytokines, MDA, Immunology, Zoology

## Abstract

*Ornithonyssus sylviarum* (*O. sylviarum*) is an obligatory, blood-sucking ectoparasite widely distributed among poultry and other mammals, causing significant economic losses. This study represented the first report of molecular genotypic identification of *O. sylviarum* from pigeons, *Columba livia domestica*, in Egypt. PCR and sequencing of the 28S rRNA gene were conducted. The resulting mite sequences were subjected to BLAST analysis, revealing 90–100% similarity to *O. sylviarum* in all tested samples. The sequences were deposited in GenBank under the accession numbers PP049086 and PP033720. A phylogenetic tree was constructed to compare the obtained species with related species worldwide. Additionally, infected pigeons showed increased expression of IL-1, IL-10, IFN-γ, and TGF-β3 genes and elevated serum levels of stress biomarkers. The increased level of these cytokines indicates there was a disturbance in the immune status of the infected host with parasite compared with control healthy ones. This increases the susceptibility to infection with other pathogens.

## Introduction

Several significant avian blood-sucking ectoparasites cause severe losses in the poultry industry and are capable of attacking various mammals, including humans^1^. Specifically, *Ornithonyssus bursa* (tropical fowl mite), *O. sylviarum* (northern fowl mite), and *Dermanyssus gallinae* (poultry red mite) are implicated in this issue. Beyond morphometrical parameters, certain epidemiological factors can aid their differentiation: *D. gallinae* is a temporary night-feeding mite, whereas *Ornithonyssus* species are permanent ectoparasites^2^.

High infections of *O. sylviarum* are associated with severe losses in poultry farms. This mite extracts approximately 6.0% of a hen’s blood per bird daily, leading to immune and behavioral stress, increased susceptibility to other pathogens, cannibalism, overall farm performance disruption, and death^1,3,4^. Avian mites are non-specific hosts, and their hungry stages can attack other hosts near their habitat. In poultry breeders, handlers, or individuals in close contact with infected birds, these parasites cause various cutaneous reactions, such as urticarial papules with or without pruritic erythematous lesions marked by a pin-point reddish plug^5^.

Accurate parasite species identification is crucial in biological studies; however, morphological identification is challenging for closely related species^6^. Molecular techniques, therefore, offer a successful and precise method of species-level identification. Among the various DNA regions commonly used as molecular identification tools, mitochondrial DNA (mit-DNA) has proven to be a specific marker for intra-specific variation and precise species-level identification due to its high mutation rate compared to the more stable nuclear DNA and its strict maternal inheritance^7,8^. Additionally, molecular markers derived from the 28S rRNA gene have been successfully utilized for the taxonomic identification of *O. sylviarum*^9^.

Mite infection in mammals enhances cell-mediated immunity (CMI), releasing pro-inflammatory cytokines and interferon^10^. The activated host immune system produces reactive oxygen species (ROS) in excessive amounts, including superoxides (O_2_^−^), hydrogen peroxides (H_2_O_2_), hydroxyl radicals (^•^OH), and nitric oxide, which function as a defense against pathogens. However, the interaction of ROS with host cells induces oxidative stress^11^. To our knowledge, no previous studies have characterized the *O. sylviarum* mite infecting pigeons in Egypt or evaluated the different pro-inflammatory cytokines in these pigeons.

Therefore, this study aimed to accurately identify the neglected *Ornithonyssus* mite species infecting pigeons in Egypt using morphological and molecular-phylogenetic tools, including PCR and sequencing of partial regions of the 28S rRNA gene. The obtained species were incorporated into a genotyping tree alongside comparable species worldwide. Additionally, pro-inflammatory cytokines such as interferon-gamma (IFN-γ), transforming growth factor-beta 3 (TGF-β3), interleukin-1 (IL-1), and interleukin-10 (IL-10) were evaluated for the first time in pigeons infected with *O. sylviarum* using quantitative real-time PCR (qRT-PCR), along with the measurement of oxidative stress markers nitric oxide (NO) and malondialdehyde (MDA).

## Materials and methods

### Ethical approval

All methods were carried out following relevant guidelines and regulations. All experimental protocols were approved by the Institutional Animal Care and Use Committee (IACUC), Faculty of Veterinary Medicine, Cairo University (Vet-CU-IACUC-08072023681) for handling pigeons and collecting samples. All methods are reported per ARRIVE guidelines.

### Sample collection

In the local pigeon market at El-Sayda-Esha, Cairo, Egypt, adult pigeons, and squabs were kept in special cages for weekly sale after being collected from their breeding places (5–10 birds per cage). Sellers and customers reported localized pruritic rashes resulting in urticarial inflammatory reactions on the legs and other body parts. Direct visual examination of these bird cages revealed the presence of fine red-colored arthropods firmly attached beneath their wings. Using brush techniques according to Rezaei et al.^12^, all available mites on the body of the infected pigeons were gently collected, kept in suitable cups, and transferred to the laboratory for preliminary identification.

Between August 2023 and January 2024, multiple visits were made to five local pigeon breeding units in El-Sayda Esha, where the previously infected pigeons were collected. After inspecting each breeding unit, mite samples were collected from all available pigeons under their wings, inside their cages, beneath manure belts, and under feed troughs. A total of 100 pigeons and their nests were investigated. All available mites on the body of the infected pigeons were gently collected using brush techniques^12^, placed in well-sealed Petri dishes, and labeled in sterile plastic tubes.

### Preparation of samples for direct microscopy

The collected samples were warmed in a water bath with 10% KOH solution for 15 min. Mites were separated from the KOH solution by centrifuging the samples at 1500 rpm for 3 min. After removing the supernatant, the sediment containing the fixed mites was gradually dehydrated through successive immersions in increasing ethanol concentrations (70%, 80%, 90%, and 100%) for 30 min each. Sediment samples were immersed in clove oil for 1 h before being transferred to xylene. Finally, the mite specimens were mounted in Canada balsam under a suitable cover slip on a glass slide. Subsequently, examinations and measurements were conducted on 40 selected samples using a micrometer slide and eyepiece, and the mean dimensions and standard deviation were calculated. Mite species were identified according to Di Palma et al.^13^.

### Molecular analysis

#### DNA extraction

A total of 25 non-engorged *O. sylviarum* samples were selected for molecular identification. The samples were grouped into collections of five mites each. Each group was washed with double-distilled water, placed in cryogenic tubes with 70% ethanol, and stored at −80 °C^4^. Subsequently, mites were removed from the ethanol, dried, and manually crushed on a sterile glass slide. The crushed mite material was transferred to an Eppendorf tube with lysing buffer using a pipette tip. Total DNA extraction was performed using the GeneJET Genomic DNA Purification Kit (Thermo Scientific, Lithuania). The extraction process included overnight digestion with proteinase K at 56 °C^14^.

#### PCR amplification and DNA sequencing

A Thermo Fisher Scientific NanoDrop ND-1000 spectrophotometer was used to assess the DNA concentration and purity. PCR amplification of a 627 bp fragment of the 28S rRNA gene sequence was performed using the forward primer (5ʹ-GCT GCG AGT GAA CTG GAA TCA AGC CT-3ʹ) and the reverse primer (5ʹ-AGG TCA CCA TCT TTC GGG TC-3ʹ), as described by^15^. The 28S rRNA gene of *O. sylviarum* was used to directly sequence the purified PCR product from each group of mites.

The PCR reactions were conducted in a 50 µL volume, with 25 µL of i-TaqTM 2× PCR master mix (Intron Biotechnology), 5 µL of DNA template, 1 µL each of forward and reverse primers, and H_2_O added to reach the final volume. The PCR assay was performed as follows: initial denaturation at 95 °C for 5 min, followed by 35 cycles of denaturation at 95 °C for 30 s, annealing at 57 °C for 30 s, and extension at 72 °C for 30 s, with a final extension at 72 °C for 10 min^16^. After electrophoresis on an agarose gel stained with ethidium bromide, the PCR amplicons were visualized using a UV transilluminator^17,18^.

Using BioEdit software, the acquired sequences were evaluated and modified. Nucleotide BLAST analysis was used to compare and align the sequences with other 28S rRNA genes of *Ornithonyssus* species available in the GenBank database (https://blast.ncbi.nlm.nih.gov/Blast.cgi). The resulting sequences were submitted to GenBank for inclusion in the GenBank database and assigned accession numbers PP049086 and PP033720. The phylogenetic analysis included only sequences obtained from the GenBank database. The phylogenetic tree was constructed using MEGA 11.0 to analyze 28S rRNA gene sequences with the neighbor-joining method^19^.

### Expression of cytokine genes in pigeons using qRT-PCR

#### RNA extraction

Total RNA was extracted from the buffy coats of 24 pigeons (12 infected and 12 noninfected) using 8.5 µL of sterile TRIzol Reagent (Invitrogen Life Technologies, Carlsbad) and 10 pmol of metabion (International AG. For the CA, USA). Two microliters of template DNA were used, following the manufacturer’s instructions. An aliquot of total RNA diluted in RNase-free water was stored to determine RNA quantity and integrity, while the remaining sample was kept at −80 °C until gene expression analysis. RNA concentration and purity were measured using a NanoDrop ND-1000 spectrophotometer (NanoDrop Technologies Inc, Delaware, USA).

#### Real-time PCR (RT-PCR)

RT-PCR was performed using the Cepheid SmartCycler^®^ II system (Sunnyvale, CA, USA) and primers listed in Table 1 to quantify the genes of IFN-γ, IL-1, TGF-β3, and IL-10 and assess gene expression in pigeons, with β-actin as the reference gene. The 25 µL reaction mixture contained 12.5 µL of SYBR green PCR master mix (Applied Biosystems, USA), 0.5 µL of each primer (10 pmol), 1 µL of cDNA (400 ng), and 10.5 µL of RNase-free water. Positive and negative controls were included for each gene of interest. Following an initial incubation at 95 °C for 5 min, 40 cycles of amplification were conducted, with denaturation at 95 °C for 30 s, annealing at 60 °C for 30 s, and extension at 60 °C for 30 s.

**Table 1:** The sets of used primers for *O. sylviarum* analysis; cytokines expression in serum of infected pigeons.

Gene	Sequence used (5′–3′)	References
Β-actin	Forward (F): AGGCTACAGCTTCACCACCAC Reverse (R): CCATCTCCTGCTCAAAATCCA	^20^
IL-1	F: CGAGAGCAGCTACGCCG R: GCCGCTCAGCACACACG	^21^
IL-10	F: TGATGAACTTAGCATCCAGCTACTC R: AACTGCATCATCTCCGACACA	^22^
IFN-γ	F: CAAGTCAAAGGCGCACGTC R: GCGTTGAGTTTTCAAGTCATTC	
TGF-β3	F: AGGACCTTGGCTGGAAATG R: ACCGTGCTGTGAGTGGTGT	

### Measurement of markers denoting oxidative stress

Malondialdehyde (MDA) and nitric oxide (NO) levels in positive and negative serum samples were measured to assess oxidative damage^23^. NO was measured according to Salem et al.^20^. The level of serum malondialdehyde (MDA) using the reaction of thiobarbituric acid and then separation occurred on HPLC. The detection was performed using UV at 532 nm. Serum nitric oxide (NO) levels were analyzed as mentioned by Khazaei and Nematbakhsh,^24^. Briefly, a colorimetric NO assay kit was used (Calbiochem–Novabiochem Corporation, San Diego, Calif), that measures the total nitrate and nitrite in serum based on the Griess reaction and measured using wavelength 450 nm as mentioned by Aktas et al.^23^ and Aytekin and Unubol Aypak^25^.

### Statistical analysis

All data were entered into PASW Statistics, version 27 software (SPSS Inc., Chicago, IL, USA)^26,27^. All statistical analyses were carried out with significance at P < 0.05. Molecular diversity indicators were reported as mean ± standard deviation (SD)^28^.

## Results

### Morphological identification

**Fig. 1:**
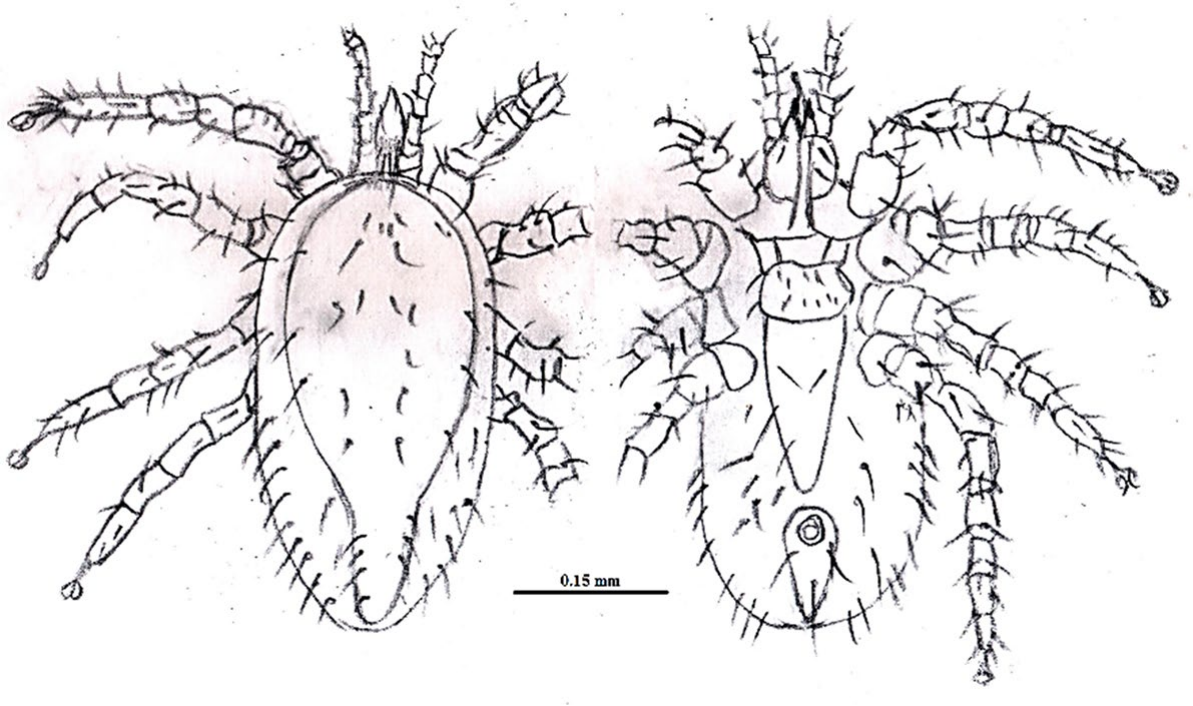
Dorsal and ventral view of *O. sylviarum* (Camera lucida drawing for mounted specimen).

Examination of male and female fed and unfed parasites collected from infected pigeons morphologically identified them as *Ornithonyssus* species. These parasites exhibit specific characteristics that differentiate them from other similar poultry mites, such as the mean dimensions of unfed parasites, which are 572 ± 10.8 × 341 ± 5.3 μm. The detailed morphological features shown in Fig. 1 supported the identification as *O. sylviarum* as the dorsal plate was wide for two-thirds of its length and then tapered to form a tongue-like continuation about half as wide for the remainder of its length. The adult mites possess two pairs of setae positioned on the ventral plate, a third pair being present on the skin immediately behind this plate or almost touching it. The genitoventral shield of the female is rounded and narrow posteriorly, and the anal opening is located at the anterior end of the anal shield. The setae on the dorsal plate like other species of poultry mite are smaller than the setae on the adjacent skin. The mites were extracted from infected pigeons during the day. For precise species identification, genotypic analysis was subsequently performed (Fig. 1).

### Molecular identification and sequencing of O. sylviarum

PCR and sequencing were performed on 25 mite samples, successfully amplifying the 28S rRNA gene in all tested mite samples. BLAST analysis of the obtained sequences confirmed absolute similarity among all samples, indicating they belonged to *O. sylviarum*. The purified PCR products yielded an approximate length of 627 bp. All nucleotide sequences were recorded in the GenBank database, and accession numbers PP049086 and PP033720 were assigned for accessibility and reference. BLAST analysis revealed that the 28S rRNA sequence was closely related to the genus *O. sylviarum*, showing the greatest similarity score (100%) to the *O. sylviarum* sequence (MT813468) from Hungary.

**Fig. 2:**
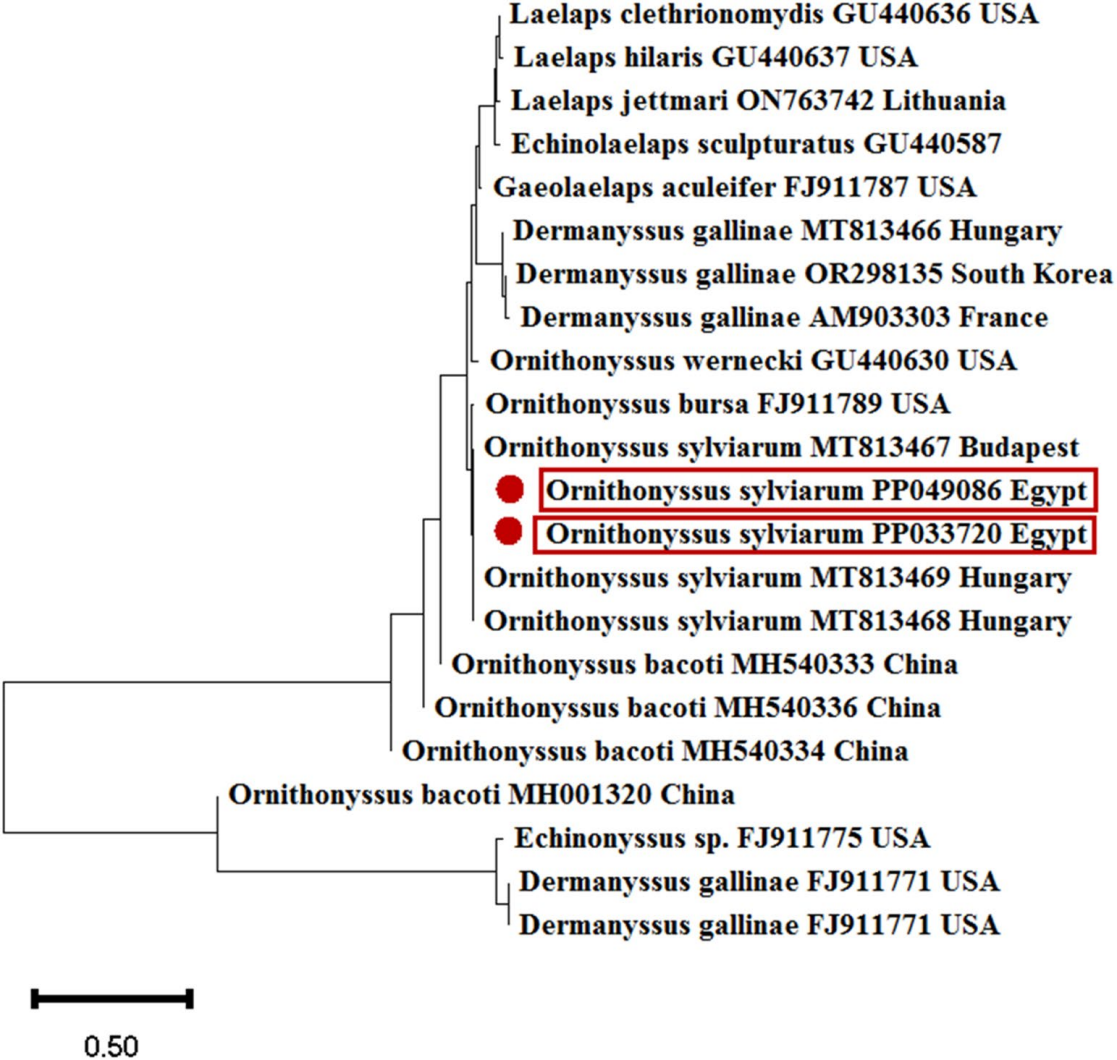
Phylogenetic analysis of *O. sylviarum* based on the 28S rRNA gene (constructed using the neighbor-joining method in MEGA 11.0).

Additionally, *O. sylviarum* sequences showed 99.88% similarity to *O. sylviarum* (MT813467) from Hungary and 98.07% and 98.85% similarity to sequences from the USA (FJ911789) and China (MH001321), respectively. Notably, the 28S rRNA sequence of *O. sylviarum* infecting pigeons in Egypt exhibited the highest identity with sequences reported in the phylogenetic tree (Fig. 2). The phylogenetic tree demonstrated strong nodal support for major clades, including *O. sylviarum*, which were distinct from other *Macronyssidae* species (Fig. 2).

### Cytokine gene expression in pigeons by qRT-PCR

**Fig. 3:**
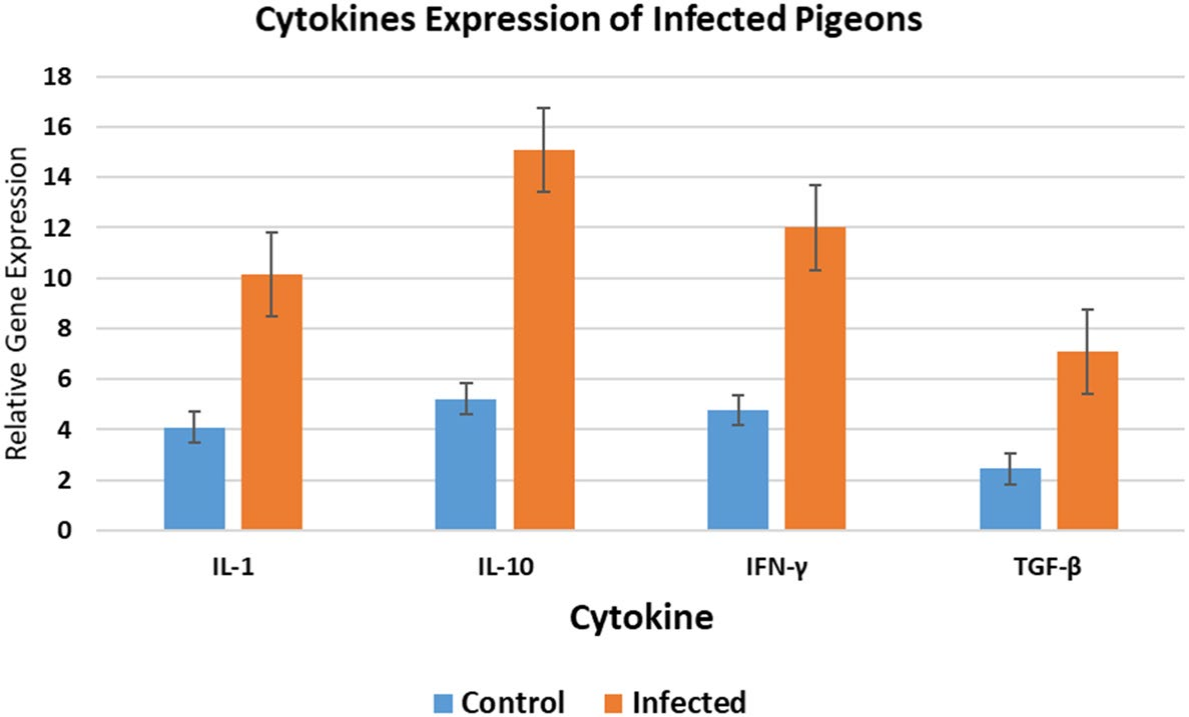
Genetic expression of various cytokines in infected pigeons.

Samples were classified into control and infected pigeons. IL-1 expression was upregulated fourfold in the control group, increasing to tenfold in infected pigeons. IL-10 was upregulated fivefold in the control group and increased to 15-fold in infected pigeons. IFN-γ transcript levels showed a fourfold upregulation in the control group, increasing to 12-fold in infected pigeons. TGF-β3 transcript levels were upregulated twofold in the control group, increasing to eightfold in infected pigeons (Fig. 3).

## Markers of oxidative stress

**Fig. 4:**
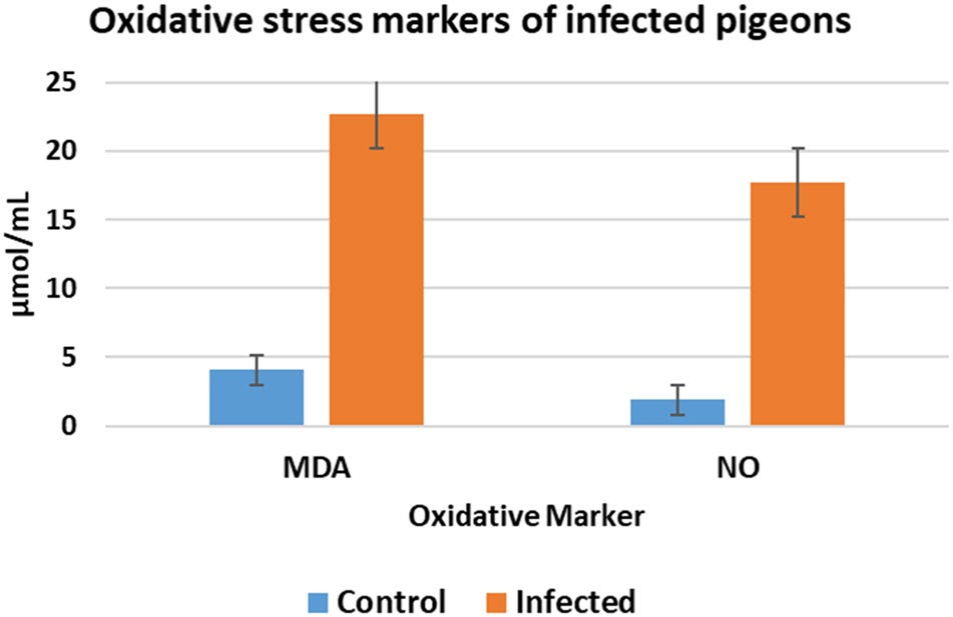
Oxidative stress markers in infected pigeons compared to control groups.

The MDA concentration in infected birds was significantly elevated compared to that in non-infected birds, reaching 22.74 ± 2.02 μmol/mL, indicating substantial oxidative stress in the infected birds. Additionally, the levels of other markers were notably higher in the infected birds, exemplified by the nitric oxide concentration, which measured 17.73 ± 1.18 μmol/L, in contrast to the control birds (Fig. 4).

## Discussion

Infections of *O. sylviarum* (known as gamasoidosis, avian mite dermatitis, and bird mite rash) affect a variety of wild and domestic birds, including ducks, finches, pigeons, starlings, psittacines, and canaries^7^. The number of reported infections of *O. sylviarum* has recently increased. Feral pigeons are particularly notable for their successful adaptations to urban environments, attributed to abundant food resources and the lack of natural predators^13^. Several reported infections have been linked to the proximity of *Columba livia domestica* (pigeons) to *O. sylviarum*, affecting patients, visiting relatives, and healthcare professionals^1^.

Accurate identification of parasite species is crucial for designing effective biological or control strategies. Morphological identification is challenging and often inefficient for differentiating closely related species due to the high levels of morphological plasticity^6^. This study is the first to accurately identify *O. sylviarum* mites infecting pigeons in Egypt through morphological, genotypic, and molecular characterization. It investigated the pro-inflammatory cytokines in both infected and control hosts and assessed the adverse impacts of the mite on the infected pigeons.

The morphological characteristics described for the obtained mites primarily identified them as *Ornithonyssus* species, based on the detailed description by^4^ and the key to mite identification used by^12,29^. Their similarity to the other two common poultry mites, the tropical fowl mite (*O. bursa*) and the poultry red mite (*D. gallinae*) were excluded as detailed by^4^, based on differences in size, the timing of host attack, number of setae on the sternal shield, position of the anal opening, and shape of the dorsal plate. According to Roy et al.^6^, morphological characteristics alone are insufficient for accurate species-level identification of parasites, making molecular tools essential for precise identification. This approach is critical for investigating potential genetic variations associated with changes in host specificity^30^. Using molecular tools, researchers can gain valuable insights into the genetic composition and potential evolutionary dynamics of *O. sylviarum* and related species.

The challenge of intraspecific morphological diversity and similarities among different mite species has led to the use of combined morpho-molecular approaches as a potential solution. 28S rRNA analysis has recently been suggested as a valuable tool for identifying certain *Acari* species. PCR amplification and sequencing techniques targeting specific genes have resolved taxonomic ambiguities in numerous parasitic species^31^.

In this study, all collected arthropods exhibited morphological compatibility, and molecular analyses of an adequate number of samples revealed identical sequences in the 28S rRNA gene region. The molecular characterizations of the mites conclusively identified them as belonging to the Macronyssidae family, specifically *O. sylviarum*. The sequence obtained in this study has been registered under accession numbers PP049086 and PP033720 for *O. sylviarum* isolated from a pigeon in Egypt. The sequence obtained from *O. sylviarum* in this study exhibited the highest similarity to sequences from Hungary (MT813468), China (MH001321), and the United States (FJ911789). This finding highlights the relatedness of *O. sylviarum* populations across different geographical locations. Furthermore, this study confirmed the value of 28S rRNA gene region sequencing and phylogenetic analysis as effective tools for differentiating and identifying *O. sylviarum*, aligning with previous reports^15,32,33^. The successful approach employed in this study for accurate genotyping of *Ornithonyssus* species in pigeons is consistent with the findings of^9^, which emphasized that molecular markers derived from the 28S rRNA gene have been successfully utilized for the taxonomic identification of *O. sylviarum* isolated from diverse hosts.

Previous literature has reported that immune system stimulation produces host protective mechanisms to combat predators. Overexpression of specific cytokines can lead to immunosuppression, allowing parasites to aggregate. The immune system’s ability to fend off *O. sylviarum* invasion has not been well studied. In this study, infection caused cellular and humoral immune responses (IL-1 and IFN-γ) linked to the host's clinical condition and the parasite’s ejection. IL-10 mRNA levels increased considerably, consistent with findings by^10,34,35^. The observed elevation in MDA concentration to 22.13 ± 3.21 μmol/mL in infected birds versus non-infected controls indicates substantial oxidative stress induced by the parasite. MDA is a well-established marker of lipid peroxidation resulting from reactive oxygen species-mediated damage^36^. The significant increase in NO levels, reaching 17.80 ± 1.12 μmol/L in infected birds compared to controls, highlights the pronounced oxidative stress caused by *Ornithonyssus* infection. Nitric oxide is a crucial immune signaling molecule produced in response to tissue damage and inflammation^37^. This rise in nitric oxide reflects an active immune response against the parasite. These findings provide valuable initial insights into the intricate parasite-host interaction in *Ornithonyssus*, setting the stage for further research. This research is crucial for mitigating the risks associated with this emerging zoonosis.

In summary, the isolates of *O. sylviarum* were genotyped, and the resulting sequence was deposited under the accession numbers PP049086 and PP033720 in the GenBank database. Identifying this mite species holds significant potential for accurate and rapid diagnosis in parasitology. Notably, this study represents the first molecular identification of *O. sylviarum* from pigeons in Giza, Egypt. Additionally, infected pigeons showed increased expression of IL-1, IL-10, IFN-γ, and TGF-β3, as well as elevated serum levels of stress biomarkers.

## Data Availability

This published article includes all the data generated and analyzed during this investigation. The datasets generated or analyzed during the current study are available in the GENBANK repository, accession numbers: [PP049086 and PP033720].

## References

[1] Murillo, A. C. & Mullens, B. A. A review of the biology, ecology, and control of the northern fowl mite, *Ornithonyssus sylviarum* (Acari: Macronyssidae). *Vet. Parasitol.* **246**, 30–37 (2017). 28969777 10.1016/j.vetpar.2017.09.002

[2] Tomley, F. M. & Sparagano, O. Spotlight on avian pathology: Red mite, a serious emergent problem in layer hens. *Avian Pathol.* **47**(6), 533–535 (2018). 29954185 10.1080/03079457.2018.1490493

[3] Eladl, A. H., Hamed, H. R. & El-Shafei, R. A. Prevalence of mites and their impact on laying hen (*Gallus gallus domesticus*) farm facilities in Egypt, with an analysis of deltamethrin residues in eggs and tissue. *Avian pathol.* **47**(2), 161–171 (2018). 28975807 10.1080/03079457.2017.1388500

[4] Bhowmick, B. *et al.* Molecular characterization and genetic diversity of *Ornithonyssus sylviarum* in chickens (*Gallus gallus*) from Hainan Island, China. *Parasites Vectors* **12**, 1–15 (2019). 31753001 10.1186/s13071-019-3809-9 PMC6873570

[5] McClain, D., Dana, A. N. & Goldenberg, G. Mite infestations. *Dermatol. Ther.* **22**(4), 327–346 (2009). 19580577 10.1111/j.1529-8019.2009.01245.x

[6] Roy, L., Dowling, A. P. G., Chauve, C. M. Lesna, I., Sabelis M. W., Buronfosse, T. Molecular phylogenetic assessment of host range in five Dermanyssus species. Control of Poultry Mites (Dermanyssus). **48**, 115–142. (2009). 10.1007/s10493-008-9231-1 19160062

[7] Jansson, D. S. *et al.* Northern fowl mite (Ornithonyssus sylviarum) in Sweden. *Med. Vet. Entomol.* **28**(4), 443–446 (2014). 24602037 10.1111/mve.12053

[8] El-Akkad, D. M. *et al.* Improved dot-ELISA assay using purified sheep coenurus cerebralis antigenic fractions for the diagnosis of zoonotic coenurosis. *Worlds Vet. J.* **12**(3), 237–249 (2022).

[9] Nieri-Bastos, F. A. *et al.* Morphological and molecular analysis of *Ornithonyssus* spp. (Acari: Macronyssidae) from small terrestrial mammals in Brazil. *Exp. Appl. Acarol.* **55**, 305–327 (2011). 21786041 10.1007/s10493-011-9475-z

[10] Blake, D., Liebhart, D. Advances in understanding parasite infections of poultry: Focus on protozoa and the red mite. In book: Optimising poultry flock health (pp. 67–104). 10.19103/AS.2022.0104.03 (2022).

[11] Shields, H. J., Traa, A. & Van Raamsdonk, J. M. Beneficial and detrimental effects of reactive oxygen species on lifespan: A comprehensive review of comparative and experimental studies. *Front. Cell Dev. Biol.* **9**, 181 (2021). 10.3389/fcell.2021.628157 PMC7905231 33644065

[12] Rezaei, F., Hashemnia, M., Chalechale, A., Seidi, S. & Gholizadeh, M. Prevalence of ectoparasites in free-range backyard chickens, domestic pigeons (*Columba livia domestica*) and turkeys of Kermanshah province, west of Iran. *J. Parasit. Dis.* **40**, 448–453 (2016). 27413319 10.1007/s12639-014-0524-5 PMC4927506

[13] Di Palma, A., Giangaspero, A., Cafiero, M. A. & Germinara, G. S. A gallery of the key characters to ease identification of *Dermanyssus gallinae* (Acari: Gamasida: Dermanyssidae) and allow differentiation from *Ornithonyssus sylviarum* (Acari: Gamasida: Macronyssidae). *Parasites Vectors* **5**(1), 1–10 (2012). 22647594 10.1186/1756-3305-5-104 PMC3419681

[14] Taha, N. M., Youssef, F. S., Auda, H. M., El-Bahy, M. M. & Ramadan, R. M. Efficacy of silver nanoparticles against *Trichinella spiralis* in mice and the role of multivitamin in alleviating its toxicity. *Sci. Rep.* **14**(1), 5843 (2024). 38462650 10.1038/s41598-024-56337-2 PMC10925591

[15] Hornok, S. *et al.* Contributions to the phylogeny of *Ixodes* (*Pholeoixodes*) *canisuga*, *I*. (*Ph*.) *kaiseri*, *I*. (*Ph*.) *hexagonus* and a simple pictorial key for the identification of their females. *Parasites Vectors* **10**(1), 1–2 (2017). 29100530 10.1186/s13071-017-2424-x PMC5670724

[16] Hornok, S. *et al.* Urban emergence of *Dermanyssus gallinae* lineage L1 and *Ornithonyssus sylviarum* in Hungary: Phylogenetic differentiation between the roles of migrating vs transported synanthropic birds. *Parasites Vectors* **14**(1), 1–9 (2021). 33685497 10.1186/s13071-021-04643-3 PMC7938540

[17] Ramadan, R. M. *et al.* Molecular and immunological studies on *Theileria equi* and its vector in Egypt. *Exp. Appl. Acarol.* **93**, 439–458 (2024). 38967736 10.1007/s10493-024-00933-4 PMC11269342

[18] Khalifa, M. M. *et al.* Dogs as a source for the spreading of enteric parasites including zoonotic ones in Giza Province, Egypt. *Res. Vet. Sci.* **161**, 122–131 (2023). 37379694 10.1016/j.rvsc.2023.06.015

[19] Salem, M. A., Mahdy, O. A., Shaalan, M. & Ramadan, R. M. The phylogenetic position and analysis of *Renicola* and *Apharyngostrigea* species isolated from Cattle Egret (*Bubulcus ibis*). *Sci. Rep.* **13**(1), 16195 (2023). 37759085 10.1038/s41598-023-43479-y PMC10533816

[20] Salem, M. A., Mahdy, O. A. & Ramadan, R. M. Ultra-structure, genetic characterization and Immunological approach of fish borne zoonotic trematodes (Family: Heterophyidae) of a redbelly tilapia. *Res. Vet. Sci.* **166**, 105097 (2024). 38007971 10.1016/j.rvsc.2023.105097

[21] Olias, P. *et al.* Modulation of the host Th1 immune response in pigeon protozoal encephalitis caused by *Sarcocystis calchasi*. *Vet. Res.* **44**, 10. 10.1186/1297-9716-44-10 (2013). 23398807 10.1186/1297-9716-44-10 PMC3598538

[22] Hayashi, T. *et al.* Host cytokine responses of pigeons infected with highly pathogenic Thai avian influenza viruses of subtype H5N1 isolated from wild birds. *PLoS One* **6**, e23103. 10.1371/journal.pone.0023103 (2011). 21826229 10.1371/journal.pone.0023103 PMC3149639

[23] Aktas, M. S., Kandemir, F. M., Kirbas, A., Hanedan, B. & Aydin, M. A. Evaluation of oxidative stress in sheep infected with using total antioxidant capacity, total oxidant status, and malondialdehyde level. *J. Vet. Res.* **61**(2), 197–201 (2017). 29978073 10.1515/jvetres-2017-0025 PMC5894390

[24] Khazaei, M. & Nematbakhsh, M. Effect of experimentally induced metabolic acidosis on aortic endothelial permeability and serum nitric oxide concentration in normal and high-cholesterol fed rabbits. *Arch. Med. Sci.* **8**(4), 719–723. 10.5114/aoms.2012.30296 (2012). 23056086 10.5114/aoms.2012.30296 PMC3460509

[25] Aytekin, I. & Unubol Aypak, S. Levels of selected minerals, nitric oxide, and vitamins in aborted Sakis pigeon raised under semitropical conditions. *Trop. Anim. Health Prod.* **43**, 511–514. 10.1007/s11250-010-9724-x.B (2012). 10.1007/s11250-010-9724-x PMC3016235 21076941

[26] Ramadan, R. M., Youssef, F. S., Fouad, E. A., Orabi, A. & Khalifa, M. M. The pharmacological impact of Astragalus membranaceus against coccidial and bacterial infection in vitro. *Egypt. Pharm. J.* **22**(2), 324–335 (2023).

[27] Taha, N. M., Sabry, M. A., El-Bahy, M. M. & Ramadan, R. M. Awareness of parasitic zoonotic diseases among pet owners in Cairo, Egypt. *Vet. Parasitol. Reg. Stud. Rep.* **51**, 101025 (2024). 10.1016/j.vprsr.2024.101025 38772640

[28] El-Bahy, M. M., Kamel, N. O., Auda, H. M. & Ramadan, R. M. A smart economic way to control camel parasites and improve camel production in Egypt. *Exp. Parasitol.* **255**, 108650 (2023). 37914150 10.1016/j.exppara.2023.108650

[29] Murthy, G. S. S. & Panda, R. Prevalence of *Dermanyssus* and *Ornithonyssus* species of mites in poultry farms of Vikarabad area of Hyderabad. *J. Parasit. Dis.* **40**(4), 1372–1375 (2016). 27876951 10.1007/s12639-015-0693-x PMC5118319

[30] Mullens, B. A., Chen, B. L. & Owen, J. P. Beak condition and cage density determine abundance and spatial distribution of northern fowl mites, *Ornithonyssus sylviarum*, and chicken body lice, *Menacanthus stramineus*, on caged laying hens. *Poult. Sci.* **89**(12), 2565–2572 (2010). 21076093 10.3382/ps.2010-00955

[31] Ramadan, R. M., Mahdy, O. A., El-Saied, M. A., Mohammed, F. F. & Salem, M. A. Novel insights into immune stress markers associated with myxosporeans gill infection in Nile tilapia (molecular and immunohistochemical studies). *PLoS One* **19**(6), e0303702 (2024). 38833454 10.1371/journal.pone.0303702 PMC11149867

[32] Dowling, A. P. & Oconnor, B. M. Phylogeny of Dermanyssoidea (Acari: Parasitiformes) suggests multiple origins of parasitism. *Acarologia* **50**(1), 113–129 (2010).

[33] Zhao, Y., Zhang, W. Y., Wang, R. L. & Niu, D. L. Divergent domains of 28S ribosomal RNA gene: DNA barcodes for molecular classification and identification of mites. *Parasites Vectors* **13**(1), 1–12 (2020). 32404192 10.1186/s13071-020-04124-z PMC7222323

[34] Steen, C. J., Carbonaro, P. A. & Schwartz, R. A. Arthropods in dermatology. *J. Am. Acad. Dermatol.* **50**(6), 819–842l (2004). 15153881 10.1016/j.jaad.2003.12.019

[35] Owen, J. P. *Interaction of the Host Immune Response and Population Dynamics of the Northern Fowl Mite, Ornithonyssus sylviarum, on White Leghorn Hens* (University of California, 2007).

[36] Razavi, S. M., Nazifi, S., Afsar, M., Yazdanpanah, Z. & Rakhshandehroo, E. Evaluation of the blood oxidant-antioxidant interactions in pigeons naturally infected with Haemoproteus columbae. *Vet. Arh.* **86**(3), 395–405 (2016).

[37] Machado-Nils, A. V. *et al.* Daily cycling of nitric oxide synthase (NOS) in the hippocampus of pigeons (*C. livia*). *J. Circadian Rhythms* **11**(1), 1–7 (2013). 24176048 10.1186/1740-3391-11-12 PMC4177212

